# Preventable Hospitalizations for Congestive Heart Failure: Establishing a Baseline to Monitor Trends and Disparities

**Published:** 2012-04-12

**Authors:** Julie C. Will, Amy L. Valderrama, Paula W. Yoon

**Affiliations:** Centers for Disease Control and Prevention, Division for Heart Disease and Stroke Prevention; Division for Heart Disease and Stroke Prevention, Centers for Disease Control and Prevention, Atlanta, Georgia; Division for Heart Disease and Stroke Prevention, Centers for Disease Control and Prevention, Atlanta, Georgia

## Abstract

**Introduction:**

Preventable hospitalization for congestive heart failure (CHF) is believed to capture the failure of the outpatient health care system to properly manage and treat CHF. In anticipation of changes in the national health care system, we report baseline rates of these hospitalizations and describe trends by race over 15 years.

**Methods:**

We used National Hospital Discharge Survey data from 1995 through 2009, which represent approximately 1% of hospitalizations in the United States each year. We calculated age-, sex-, and race-stratified rates and age- and sex-standardized rates for preventable CHF hospitalizations on the basis of the Agency for Healthcare Research and Quality's specifications, which use civilian population estimates from the US Census Bureau as the denominator for rates.

**Results:**

Approximately three-fourths of the hospitalizations occurred among people aged 65 years or older. In each subgroup and period, rates were significantly higher (*P* < .05) for blacks than whites. Only black men aged 18 to 44 showed a linear increase (*P* = .004) in crude rates across time. Subpopulations aged 65 or older, except black men, showed a linear decrease (*P* < .05) in crude rates over time. Age- and sex-standardized rates showed a significant linear decrease in rates for whites (*P* = .01) and a borderline decrease for blacks (*P* = .06)

**Conclusion:**

Before implementation of the Patient Protection and Affordable Care Act, we found that blacks were disproportionately affected by preventable CHF hospitalizations compared with whites. Our results confirm recent findings that preventable CHF hospitalization rates are declining in whites more than blacks. Alarmingly, rates for younger black men are on the rise.

## Introduction

Studies and expert committees have identified conditions for which many hospitalizations could be avoided if patients received early access to good-quality health care (1,2). These conditions have been labeled ambulatory-care–sensitive conditions (ACSCs) (1,2) and are used by the federal government as Prevention Quality Indicators (PQIs) (3). Congestive heart failure (CHF) is one such ACSC (3).

Clinical guidelines are available for the diagnosis and management of CHF (4,5), and evidence exists that physicians could better adhere to these guidelines (6). Other ways to reduce the likelihood of hospitalization are disease management programs (eg, increased follow-up) and self-management programs including symptom monitoring, weight monitoring, or medication dosage adjustment (7,8).

Prevention of future hospitalizations can occur even before symptoms of CHF occur. Evidence-based strategies in the outpatient setting for these patients include control of hypertension and low-density lipoprotein cholesterol, use of angiotensin-converting enzyme inhibitors if appropriate, smoking cessation, physical activity, weight management, and diabetes management (4,9).

The 2010 Patient Protection and Affordable Care Act (PPACA) aims to increase access to outpatient care and improve the quality of such care through implementation of evidence-based outpatient management systems and strategies (10). We used national hospital survey data to describe pre-PPACA baseline rates of preventable CHF hospitalizations and describe racial differences in these rates over time. Although others have examined similar trends over time (11,12), 1 group of researchers recently reported a decline in national CHF hospitalization rates for blacks and whites aged 65 years or older (11). We extend these recent findings by examining more years of data and by examining both younger and older adults. We anticipate our results being useful now and in the future to help monitor the effect of changes in the national health care system.

## Methods

### Data source and definitions

We used data from the 1995 through 2009 National Hospital Discharge Survey (NHDS) conducted by the National Center for Health Statistics (NCHS) (13). Details of the sampling design for each year of the study are provided on the NCHS website (www.cdc.gov/nchs/nhds/nhds_sample_design.htm). For the 1995 through 2007 data, the sample included 501 to 525 hospitals; the average unweighted response rate was 89%. In 2008 and 2009, NCHS had only enough funding to collect data from 239 hospitals; the unweighted response rates were 86% to 87%. At the final stage of sampling, the NHDS selects a systematic random sample of inpatient discharge records from each participating hospital, representing approximately 1% of all hospitalizations in the United States.

We calculated population-based hospital discharge rates according to specifications published by the Agency for Healthcare Research and Quality (AHRQ) for PQI #8, congestive heart failure admission rate (14). To be defined as a preventable hospitalization, the numerator consists of all nonmaternal discharges of people aged 18 years or older with the following *International Classification of Diseases, Ninth Revision, Clinical Modification* (ICD-9-CM) principal diagnosis codes: 398.91, 428.0, 428.1, and 428.9. Because of ICD-9-CM coding changes in 2002, AHRQ requires that principal diagnosis codes of 402.01, 402.11, 402.91, 404.01, 404.03, 404.11, 404.13, 404.91, and 404.93 be included in the numerator only through September 30, 2002. After that, AHRQ requires that the following principal diagnoses codes be included in the numerator: 428.20, 428.21, 428.22, 428.23, 428.30, 428.31, 428.32, 428.33, 428.40, 428.41, 428.42, and 428.43. Specifications require that maternal discharges identified using major diagnostic category 14 (pregnancy, childbirth, and puerperium) be excluded from the numerator. The NHDS data are not grouped by major diagnostic codes; thus, maternal discharges were identified according to another method that uses ICD-9-CM codes alone (15). AHRQ further specifies that preventable hospitalizations for CHF exclude from the numerator transfers from another institution including a hospital, skilled nursing facility, or intermediate care facility (database missing this information before 2001) and discharges with cardiac procedure codes in any field. Excluded were people hospitalized with procedures such as implants and maintenance of pacemakers, surgical procedures for repair of heart valves, revascularization, coronary bypass surgery, heart transplants, and other such procedures. Because of these exclusions, the most serious cases of CHF were eliminated, leaving those hospitalizations that are believed to be most preventable. People who received diagnostic testing (eg, echocardiograms, catheterization, stress testing) or who received nonsurgical treatments (eg, diuresis, medication adjustments) were included in the numerator. Eligible hospitalizations defined as above will hereafter simply be called hospitalizations for CHF.

The denominators for the rates are the civilian population estimates from the US Census that are published by NCHS as part of the documentation package for each year's NHDS database (13).

We used the confidential database for this analysis, which allowed us to use sampling design variables to calculate standard errors around estimates. The descriptor of primary interest was race, which was reported for approximately 80% of the records. We used 2 categories of race for this analysis: white or black (we did not separate out whether someone was Hispanic because of the large number of discharges that were missing this information) (16). We also created an "other" category that consisted of Asian, American Indian, Alaska Native, Eskimo, Native Hawaiian, Pacific Islander, and any other race that was specified on the form. Race was considered missing if the medical record abstract form had "not stated" checked or if multiple races were recorded. The multiracial category was included in the missing category because it has only been coded in the database since 2000 and accounts for a small percentage of hospitalizations (eg, during 2005-2006 it was 3%). Although we used the "other" category in a description of hospitalizations for CHF, we did not use it for our main analysis because the sample sizes were small with broad confidence intervals, especially after stratification by age and sex. The geographic areas included in the study were the 4 census regions: Northeast, Midwest, South, and West (17). For insurance type, we used Medicare, Medicaid, private insurance, and other. We used only the principal source of payment to derive these categories. "Other" included types such as other government insurance, self-pay, hospitalization without a charge, and workers' compensation.

### Statistical analysis

We estimated the total weighted number of hospitalizations for CHF each year from 1995 through 2009 for people aged 18 or older. Because we analyzed only records for which CHF was the primary reason for the hospitalization and because we were interested in studying a smaller subgroup of the population (ie, blacks), we combined 2 years of data to obtain a larger number of reliable estimates for 1995 through 2006. Because half samples were used in 2008 and 2009, we combined 3 years of data to obtain reliable estimates for 2007 through 2009. We combined the smallest number of years to achieve relative standard errors that were smaller than 30% for each age-race-sex stratum. Consequently, we had 7 analytic periods (1995-1996, 1997-1998, 1999-2000, 2001-2002, 2003-2004, 2005-2006, and 2007-2009). We summed census population estimates over these same periods and used them to calculate rates per 100,000 population.

Because we were interested in describing the burden of hospitalizations in various population subgroups, we report crude rates by age (18-44, 45-64, and ≥65), sex, and race. We also directly standardized rates for each period and racial group by age and sex using the 2000 Census population as the standard.

We used SUDAAN (Research Triangle Institute, Research Triangle Park, North Carolina) to calculate 95% confidence intervals around the estimates of the number of hospitalizations for CHF. We did not develop confidence intervals around the denominators because they were derived from a census of the population. We tested differences between subgroups using *z* tests; significance was set at *P* < .05. We used simple linear regression, weighted by the inverse of the variance of each year's rate, to test for linear time trends for each study subgroup and for the standardized rates. We used the *t* test to determine whether the slope was significantly different from zero. We did not test for nonlinear trends because of the complexity of interpreting curves with a restricted number of points and the difficulty of explaining transformed rate data.

## Results

During 1995 through 2009, there were 121,741 records with preventable hospitalizations for CHF among adults in the NHDS sampled hospitals. This translates to a weighted number of 15,208,518 hospitalizations for adults in the United States during the 15-year study period, an average of 1,013,901 each year.

Approximately three-fourths of preventable hospitalizations for CHF were among people aged 65 or older (Table 1). In 2007 through 2009, as in every other period, a higher percentage of these hospitalizations occurred for women (53%) than for men (47%). The South had the highest percentage of hospitalizations (37%-39%). The West had less than 15% of hospitalizations. The most frequent payer was Medicare, ranging from 73% to 77%, depending on the period. No other payer came close to this percentage; for example, in 2007 through 2009, Medicaid paid for 8%, private insurance paid for 13%, and all other types of coverage accounted for 5%.

For women, the crude rates for preventable CHF hospitalizations increased with age for both racial groups in each period (Table 2). In all subgroups of women, the crude estimates for black women were higher than those for white women during all 7 periods (Table 2). For white women, we found a significant decreasing linear trend for those aged 45 through 64 and for those aged 65 or older. For black women, we found a decreasing linear trend for those aged 65 or older (Table 2). For white and black women, the highest rate occurred among those aged 65 or older; it peaked in 1997 through 1998 for whites (1,903.8/100,000) and in 1999 through 2000 for blacks (3,303.6/100,000).

Crude rates for preventable CHF hospitalizations among men increased with age during every period (Table 3). In all subgroups of men, the point estimates for black men were significantly larger than for white men (Table 3). We found an increasing linear trend in rates for black men aged 18 to 44. For white men aged 65 or older we found a decreasing linear time trend (Table 3). For white and black men, the highest rate occurred among those aged 65 or older; it peaked in 1997 through 1998 for whites (1,932.6/100,000) and in 1995 through 1996 for blacks (2,706.5/100,000). For both women and men, the ratio of the rates for blacks compared to the rates for whites was highest in the youngest age group (~6.5) and lowest in the oldest age groups (~1.5).

Age- and sex-standardized rates also show that blacks had higher rates of hospitalization for CHF than did whites (Figure). The negative linear trend for whites was significant and the negative trend for blacks was of borderline significance. The slopes for whites and blacks were not significantly different from each other.

**Figure.**
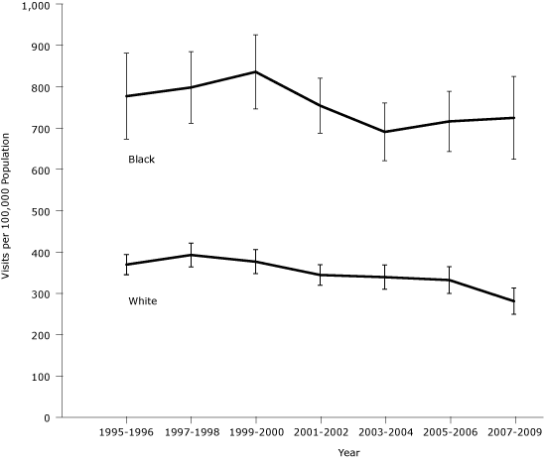
Age- and sex-standardized preventable hospitalization rates for congestive heart failure in the United States across 7 periods. Error bars represent 95% confidence intervals. There was a negative linear trend for whites (β = −14/100,000; *P* = .01) and a borderline decrease for blacks (β = −19/100,000; *P* = .06). The slopes for blacks and whites were not significantly different from each other. Source: National Hospital Discharge Survey, 1995-2009.

**Table:** 

Race	Visits per 100,000 (95% Confidence Interval)						

	1995-1996	1997-1998	1999-2000	2001-2002	2003-2004	2005-2006	2007-2009
White	369.0 (344.8-393.2)	392.3 (363.8-420.8)	376.3 (347.3-405.3)	344.0 (319.0-369.0)	338.9 (309.5-368.5)	331.7 (299.3-364.1)	280.8 (248.9-312.7)
Black	775.9 (672.1-879.7)	797.1 (710.4-883.8)	834.8 (745.3-924.3)	753.0 (686.7-819.3)	689.7 (620.0-759.4)	715.1 (642.3-787.9)	723.7 (623.9-823.5)

## Discussion

We studied hospital discharges from 1995 through 2009 and found that blacks in every age and sex subgroup had significantly higher rates of hospitalization for CHF than did whites. For both women and men, the ratio of the rates for blacks compared to the rates for whites was highest in the youngest age group (~6.5) and lowest in the oldest age groups (~1.5). Our results generally confirm recent findings (11) demonstrating a significant declining linear trend in CHF hospitalizations for people aged 65 or older. However, we did not find that black men in this age group experienced these same declining rates. In fact, we found a significant increasing linear trend for black men aged 18 to 44.

Other investigators have reported racial differences in preventable CHF hospitalizations. Using hospital data from 1991 through 1998 in California for people aged 20 to 64, Davis and colleagues found that non-Hispanic black men and women had unadjusted rates that were 4.1 and 5.5 times higher, respectively, than those for their non-Hispanic white counterparts (18). Using 1997 data from 22 states, Laditka and others studied a similarly aged population of non-Hispanics and found that black men were 3.4 times as likely to be hospitalized for CHF as white men, and black women were 6.5 times as likely to be hospitalized as white women (19). In 2003, Russo et al studied hospitalizations for people aged 18 or older from 23 states and found that age- and sex-adjusted rates for non-Hispanic black men and women combined were 2.5 times higher than those for non-Hispanic white men and women (20). Using Medicare data from Maryland in 2006, O'Neill and colleagues (21) found that adjusted rates for blacks were 60% higher than for whites. Using the 1995 through 2004 NHDS but with a different case definition than in our study, Zhang and Watanabe-Galloway found that blacks aged 65 or older had higher CHF hospitalization rates than did whites (12).

Among adults aged 45 to 84, blacks have approximately twice the incidence of CHF as whites (22), and blacks with CHF may have more comorbidities such as uncontrolled high blood pressure (23,24). This higher incidence and comorbidities may be reflected in the higher hospitalization rates among blacks (24). Furthermore, blacks, when affected by heart failure, experience a unique epidemiology and natural history (ie, disease occurring at an earlier age resulting in more substantial left ventricular dysfunction), which also may contribute to these increased rates (25). The cost of medications and type of insurance may create barriers to appropriate care, for example, by leading to the underuse of dietary and medication therapies (26-28). Blacks are less likely than whites to receive medical care such as appropriate diagnostic procedures, thrombolytic therapy, and revascularization procedures for acute coronary syndrome (29), and these delays in receiving quality care could help explain the higher hospitalization rates for blacks.

Our study contributes to the literature in the following ways: 1) we confirm a declining linear trend in CHF hospitalizations in much of the older population using data from hospitals sampled throughout the 50 states and the District of Columbia, 2) we provide national age- and sex-specific rates for multiple periods, thus establishing a baseline for future monitoring of health disparities, 3) we provide confidence intervals (calculated appropriately from a confidential database that includes design variables) showing the degree of certainty or uncertainty of point estimates and allowing judgments about the significance of differences between subpopulations, and 4) our results cover all adults and extend through 2009.

Our study also has several limitations. First, race was not reported for 17% to 21% of the CHF hospitalizations. These missing values likely are disproportionately occurring in the white population because hospitals that did not report race had a higher proportion of white discharges than hospitals that did; thus, our results may overestimate the differences in rates between blacks and whites (16). We believe that because the differences between racial groups are already large, it is unlikely that the distribution would be so skewed as to eliminate the differences between the 2 groups (16). For example, using data from 2005 through 2006, and assigning all missing hospitalizations to whites, we observed an increase in the rate for whites (from 353/100,000 to 461/100,000). Even after such an extreme assumption, blacks still have a higher rate (601/100,000) than whites. Also, because our findings are supported by many other researchers (12,19-22), we believe that these differences are likely real. Second, starting in 2000, NCHS allowed abstractors to record multiple races and to record them as such in the database, likely leading to some people who would have been classified before 2000 as either black or white now being classified as multiracial. Because only 3% of the records have race classified as multiracial, the effect is likely minimal. The difference between blacks and whites is smaller in our study than in previously published studies, most likely because we did not exclude all Hispanics from our groups of blacks and whites (other studies used the classification of non-Hispanic white compared to non-Hispanic black). Finally, as required by AHRQ's definition of a PQI, we were unable to exclude CHF hospitalizations that occurred because of transfers from another facility (ie, hospital, skilled nursing facility, or intermediate health facility) for the years 1995 through 2000 because transfer data were not collected during those years. However, on the basis of the years for which transfer data were available, we believe that excluding them from 2001 through 2009 created only a small error, given that transfers accounted for approximately 3% of CHF hospitalizations.

Our results and those of others indicate that CHF hospitalizations are higher among blacks than whites. It also appears that rates are dropping in a linear fashion for whites, mainly because of decreasing rates in the population aged 65 or older. It is alarming that in most subpopulations of blacks, rates are either remaining level or are increasing; most disturbing is the increasing linear trend for younger black men. Primary care strategies such as heart failure disease management programs (8) and aggressive comprehensive risk factor management (4,9) may help close this gap between blacks and whites and younger and older Americans. We advocate for continuing surveillance of these trends and suggest that these preventable hospitalizations may be a useful metric for monitoring changes associated with health care reform. In addition, further studies aimed at examining the potential reasons for such racial differences are needed. These studies will likely require merging data from various data sources and using multivariate analyses.

## Figures and Tables

**Table 1.** Preventable Hospitalizations for Congestive Heart Failure, by Selected Demographic Characteristics, National Hospital Discharge Survey, United States, 1995-2009

Characteristic	Year, % (95% CI)						

	1995-1996 (n = 1,935,767)	1997-1998 (n = 2,132,250)	1999-2000 (n = 2,178,509)	2001-2002 (n = 2,040,211)	2003-2004 (n = 2,057,366)	2005-2006 (n = 2,045,365)	2007-2009 (n = 2,819,050)
**Age, y**							
18-44	3.5 (2.9-4.1)	3.0 (2.5-3.5)	3.5 (2.9-4.2)	3.1 (2.7-3.6)	3.5 (2.9-4.2)	4.1 (3.5-4.8)	3.9 (3.3-4.6)
45-64	17.28 (16.1-18.5)	18.2 (16.9-19.5)	18.6 (17.5-19.8)	21.8 (20.4-23.3)	22.2 (20.7-23.8)	21.7 (20.1-23.4)	23.0 (21.3-24.6)
≥65	79.3 (78.0-80.5)	78.9 (77.4-80.2)	77.9 (76.6-79.1)	75.0 (73.5-76.5)	74.3 (72.6-75.9)	74.3 (72.4-76.0)	73.2 (71.3-75.0)
**Sex**							
Male	43.0 (41.4-44.8)	44.0 (42.3-45.8)	42.2 (40.9-43.4)	44.5 (43.2-45.8)	45.2 (43.8-46.7)	45.3 (42.9-47.6)	46.7 (45.2-48.3)
Female	57.0 (55.3-58.7)	56.0 (54.2-57.7)	57.8 (56.6-59.1)	55.5 (54.2-56.8)	54.8 (53.3-56.2)	54.7 (52.4-57.1)	53.3 (51.7-54.8)
**Race^a^**							
White	79.6 (76.2-82.6)	79.0 (76.1-81.6)	77.7 (74.4-80.7)	77.5 (74.6-80.2)	78.3 (75.0-81.2)	77.6 (74.4-80.6)	73.8 (69.4-77.7)
Black	16.9 (14.2-20.1)	16.8 (14.5-19.4)	18.5 (15.8-21.7)	18.7 (16.3-21.4)	17.9 (15.3-20.8)	19.3 (16.6-22.4)	22.3 (18.7-26.4)
Other	3.5 (2.3-5.2)	4.2 (3.3-5.5)	3.7 (2.8-5.0)	3.8 (2.8-5.2)	3.8 (2.8-5.3)	3.0 (2.1-4.4)	3.9 (2.9-5.3)
**Region**							
Northeast	23.3 (20.9-25.9)	22.0 (19.9-24.2)	23.6 (20.6-27.0)	23.3 (20.1-26.8)	22.3 (19.0-26.1)	22.5 (18.4-27.2)	22.7 (17.5-29.0)
Midwest	25.7 (22.7-28.8)	26.9 (23.7-30.3)	25.4 (22.0-29.2)	25.3 (21.9-29.0)	25.0 (21.6-28.4)	25.1 (21.5-29.1)	23.8 (18.6-29.9)
South	37.6 (34.5-40.8)	37.4 (33.9-41.0)	38.5 (34.9-42.2)	37.2 (34.1-40.5)	39.0 (35.8-42.3)	38.0 (34.4-41.8)	39.4 (32.2-47.1)
West	13.5 (11.6-15.6)	13.8 (12.3-15.4)	12.5 (11.0-14.2)	14.2 (12.2-16.4)	13.8 (11.5-16.5)	14.5 (12.5-16.6)	14.1 (9.9-19.7)
**Insurance coverage^b^**							
Medicare	76.5 (74.4-78.6)	75.7 (74.0-77.3)	75.6 (73.7-77.3)	74.1 (72.4-75.7)	74.6 (72.8-76.3)	73.0 (71.2-74.8)	74.4 (72.4-76.3)
Medicaid	5.8 (5.1-6.6)	5.1 (4.5-5.8)	5.8 (5.2-6.6)	6.8 (5.9-7.8)	8.3 (7.2-9.6)	7.9 (6.9-9.2)	7.9 (6.8-9.3)
Private insurance	13.4 (11.6-15.4)	14.9 (13.6-16.2)	15.2 (13.8-16.7)	15.1 (13.8-16.6)	13.1 (12.0-14.4)	13.7 (12.3-15.3)	12.9 (11.6-14.2)
All other	4.3 (3.6-5.1)	4.3 (3.7-5.1)	3.4 (2.9-4.0)	4.0 (3.3-4.8)	4.0 (3.4-4.6)	5.3 (4.6-6.2)	4.8 (4.1-5.7)

Abbreviation: CI, confidence interval.

a Totals are less than totals for the other characteristics because of missing values: 327,422 (16.9%) in 1995-1996; 377,994 (17.7%) in 1997-1998; 428,915 (19.7%) in 1999-2000; 395,890 (19.4%) in 2001-2002; 422,471 (20.5%) in 2003-2004; 393,172 (19.2%) in 2005-2006; and 516,833 (18.3%) in 2007-2009.

b Totals are less than totals for the other characteristics because of missing values: 23,591 (1.2%) in 1995-1996; 18,101 (0.8%) in 1997-1998; 15,044 (0.7%) in 1999-2000; 26,372 (1.3%) in 2001-2002; 28,155 (1.4%) in 2003-2004; 25,687 (1.3%) in 2005-2006; and 47,240 (1.7%) in 2007-2009.

**Table 2.** Crude Preventable Hospitalization Rates for Congestive Heart Failure per 100,000 Population Among Women, by Age and Race, National Hospital Discharge Survey, United Stated, 1995-2009

Age/Race	Year, n (95% CI)						

	1995-1996	1997-1998	1999-2000	2001-2002	2003-2004	2005-2006	2007-2009
**18-44 y**							
White	15.0 (7.8-22.1)^a^	9.2 (5.5-12.8)^a^	10.6 (6.2-15.0)^a^	10.5 (5.5-15.6)^a^	14.7 (8.7-20.7)^a^	10.5 (6.0 15.0)^a^	8.5 (4.1-12.9)^a^
Black	77.0 (48.7-105.3)	98.0 (60.1-135.8)	111.8 (48.4-175.2)	86.4 (63.6-109.3)	64.1 (45.9-85.2)	70.0 (47.5-92.5)	104.9 (62.9-146.8)
**45-64 y**							
White	169.5 (139.9-199.1)^a^	188.9 (149.9-227.9)^a^	178.1 (142.8-213.4)^a^	178.4 (145.7-211.1)^a^	167.5 (135.1-199.9)^a^	150.9 (120.2-181.6)^a^	129.3 (101.5-157.0)^a^^,^ b
Black	736.2 (609.4-863.1)	876.13 (705.7-1046.6)	917.0 (735.6-1098.4)	824.5 (684.9-964.1)	741.8 (562.6-921.0)	671.8 (559.4-784.2)	689.6 (501.6-877.6)
**≥65 y**							
White	1,835.7 (1,647.0-2,024.3)^a^	1,903.8 (1,680.9-2,126.8)^a^	1,892.2 (1,679.8-2,104.7)^a^	1,616.9 (1,429.4-1,804.3)^a^	1,606.8 (1,386.2-1,827.5)^a^	1,646.2 (1,372.3-1,920.1)^a^	1,300.1 (1,060.9-1,539.3)^a^^,^ c
Black	2,819.4 (1,939.4-3,699.5)	2,983.4 (2,404.7-3,562.2)	3,303.6 (2,614.9-3,992.2)	2,734.0 (2,241.3-3,226.8)	2,377.3 (1,899.7-2,854.9)	2,434.9 (1,920.4-2,949.4)	2,475.6 (1,862.6-3,088.7)^d^

Abbreviation: CI, confidence interval.

a Difference between white and black is significant at *P* < .05. Calculated by using a z test.

b Decreasing linear trend is significant (as determined by a *t* test of β = 0) at *P* = .03.

c Decreasing linear trend is significant (as determined by a *t* test of β = 0) at *P* = .01.

d Decreasing linear trend is significant (as determined by a *t* test of β = 0) at *P* = .049.

**Table 3.** Crude Preventable Hospitalization Rates for Congestive Heart Failure per 100,000 Population Among Men, by Age and Race, National Hospital Discharge Survey, United Stated, 1995-2009

Age/Race	Year, n (95% CI)						

	1995-1996	1997-1998	1999-2000	2001-2002	2003-2004	2005-2006	2007-2009
**18-44 y**							
White	18.9 (11.4-26.3)^a^	16.3 (10.8-21.8)^a^	14.3 (8.4-20.3)^a^	14.7 (8.5-21.0)^a^	17.6 (10.0-25.3)^a^	24.8 (16.6-32.9)^a^	12.6 (7.2-18.1)^a^
Black	101.1 (56.7-145.6)	92.6 (61.2-124.1)	118.7 (79.3-158.0)	125.2 (92.7-157.7)	145.9 (93.0-199.0)	159.6 (112.7-206.4)	143.0 (91.3-194.7)^b^
**45-64 y**							
White	209.3 (169.2-249.4)^a^	236.9 (197.4-276.5)^a^	215.4 (184.3-246.6)^a^	238.4 (199.8-277.1)^a^	231.4 (187.9-274.8)^a^	215.0 (174.6-255.3)^a^	187.6 (147.4-227.8)^a^
Black	1,024.8 (799.5-1,250.1)	915.9 (659.9-1,172.0)	895.3 (720.1-1,070.4)	974.7 (798.6-1,150.8)	796.0 (649.6-942.4)	977.2 (792.7-1,161.8)	911.6 (692.9-1,130.3
**≥65 y**							
White	1,762.4 (1,572.2-1,952.7)^a^	1,932.6 (1,702.2-2,163.0)^a^	1,788.1 (1,519.9-2,056.3)^a^	1,665.8 (1,450.9-1,880.7)^a^	1,617.3 (1,358.8-1,875.8)^a^	1,518.3 (1,289.5-1,747.1)^a^	1,435.9 (1,147.2-1,724.6)^a^^,^ c
Black	2,706.5 (2,137.4-3,275.6)	2,648.8 (2,002.7-3,294.9)	2,536.2 (1,968.1-3,104.3)	2,278.5 (1,872.5-2,684.6)	2,438.5 (1,941.9-2,935.1)	2,422.1 (1,861.4-2,982.7)	2,516.5 (1,726.1-3,307.0)

Abbreviation: CI, confidence interval.

a Difference between white and black is significant at *P* < .05. Calculated by using a z test.

b Increasing linear trend is significant (as determined by a *t* test of β = 0) at *P* = .004.

c Decreasing linear trend is significant (as determined by *t* test of β = 0) at *P* = .01.
